# Unusual Cause of Stroke

**DOI:** 10.7759/cureus.11517

**Published:** 2020-11-17

**Authors:** Mariem Borcheni, Emad Kandah, Basel Abdelazeem, Saed Alnaimat, Arvind Kunadi

**Affiliations:** 1 Internal Medicine, University of Sfax, Sfax, TUN; 2 Internal Medicine, McLaren Health Care, Michigan State University, Flint, USA; 3 Cardiology, McLaren Health Care, Michigan State University, Flint, USA

**Keywords:** atrial myxoma, cardiac tumor, cardioembolic stroke, stroke, echocardiogram

## Abstract

Every year, more than 795,000 people in the United States have a stroke, the vast majority of which are ischemic. Cardiac myxoma is an unusual cause of stroke and accounts for less than 1% of ischemic strokes. We present a case of a 56-year-old male with a history of hypertension, dyslipidemia, and type 2 diabetes mellitus, who presented with altered mental status, tinnitus, double vision, and diaphoresis. Due to concern for a cerebral vascular accident, a CT scan of the brain was obtained and showed no acute intracranial process. Brain MRI revealed multiple small acute infarcts involving bilateral posterior cerebral artery distribution. Further evaluation included transthoracic echocardiography that showed a large mobile mass in the left atrium measuring 3.5 x 2 cm intermittently projecting through the mitral valve. The patient underwent successful surgical resection of the left atrial mass. The pathology report confirmed the diagnosis of atrial myxoma. This case further highlights the importance of complete evaluation of stroke, including echocardiography, as well as the importance of careful surgical resection to prevent recurrence of systemic embolization and other complications of atrial myxoma.

## Introduction

Acute stroke is the fifth leading cause of death in the United States and a major health hindrance [1]. The vast majority of acute strokes are ischemic. Nearly one-fourth of ischemic strokes are caused by a cardioembolic phenomenon, primarily due to atrial fibrillation [2]. Cardiac myxomas account for less than 1% of ischemic strokes [3]. Despite being the most common cardiac tumor, myxoma is a very rare occurrence, with an incidence rate of 0.5 per million population per year [4,5]. Cardiac myxomas are associated with a triad of complications, including obstructive, embolic, and constitutional complications. Acute cerebral infarction may be the first manifestation of left atrial myxoma in one-third of cases [6].

## Case presentation

**Figure 1:**
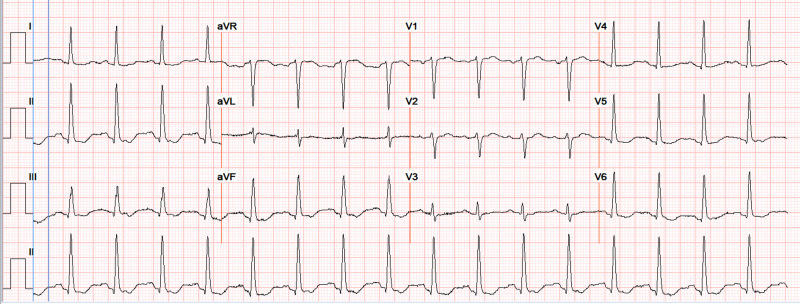
Electrocardiogram showed sinus tachycardia and T wave inversion in inferolateral leads

A 56-year-old male with a history of hypertension, dyslipidemia, and type 2 diabetes mellitus presented to the emergency department with sudden onset of tinnitus, double vision, diaphoresis, and altered mental status. The patient also reported diffuse headache in the occipital region. He had a blood pressure of 177/104 mmHg, pulse of 119 bpm, temperature of 98.2 F, respiratory rate of 16 rpm, and oxygen saturation of 97% on room air. On physical examination, the patient was awake and alert, pupils were equal and reactive. He had no nystagmus or focal neurologic deficits. Cardiovascular examination showed tachycardia. His examination was otherwise unremarkable. Electrocardiogram showed sinus tachycardia and T wave inversion in inferolateral leads (Figure 1). Laboratory workup showed a slightly elevated white blood cell count of 12.04 10*3/uL (reference range 4.50-10.00 x 10*3/uL). Troponin-I, thyroid stimulating hormone (TSH), brain natriuretic peptide, and ammonia levels were all normal.

**Figure 2:**
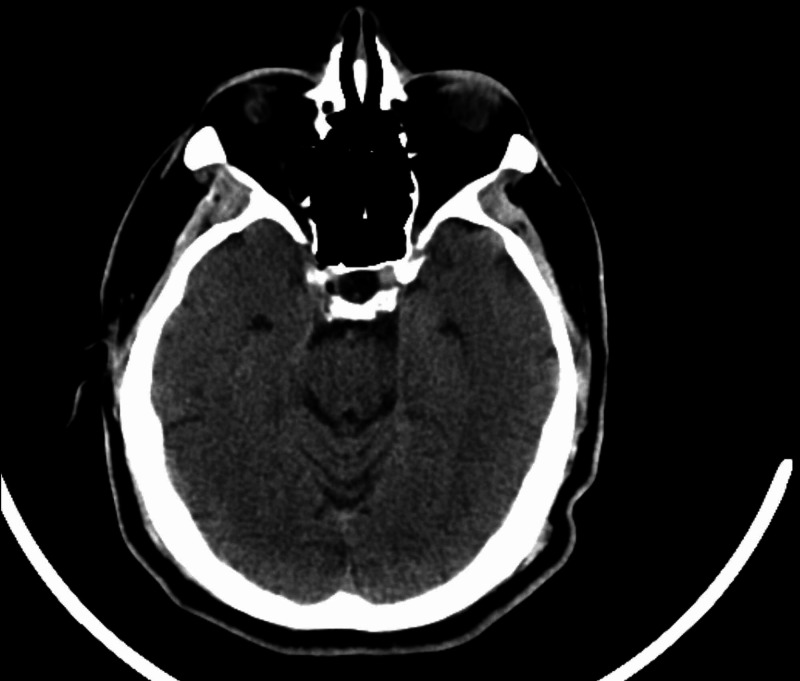
Computed tomography of the brain without contrast showed no signs of intracranial hemorrhage or brain infarction

**Figure 3:**
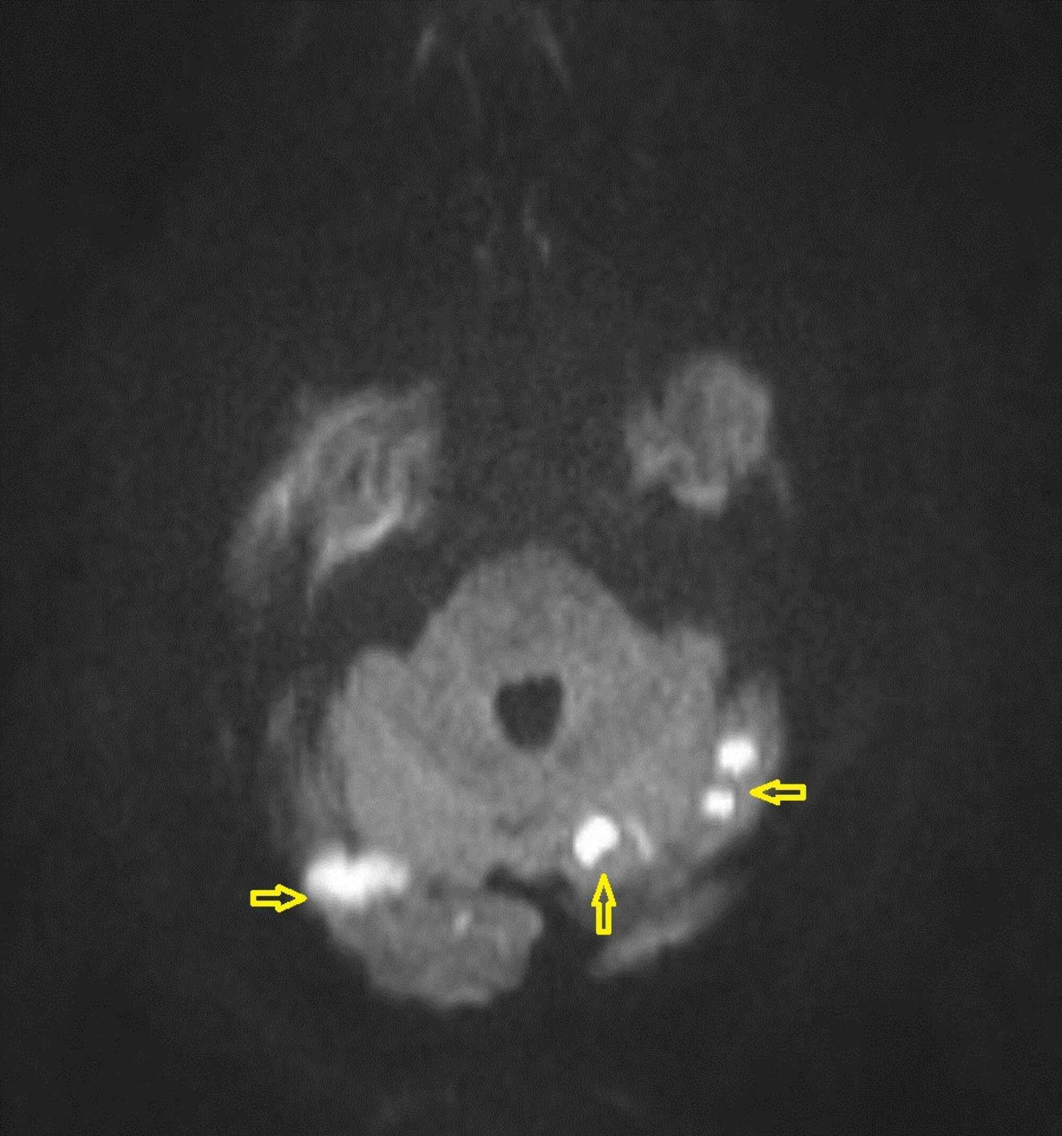
MRI Brain showed multiple small acute infarcts involving bilateral posterior cerebral arteries distribution

**Figure 4:**
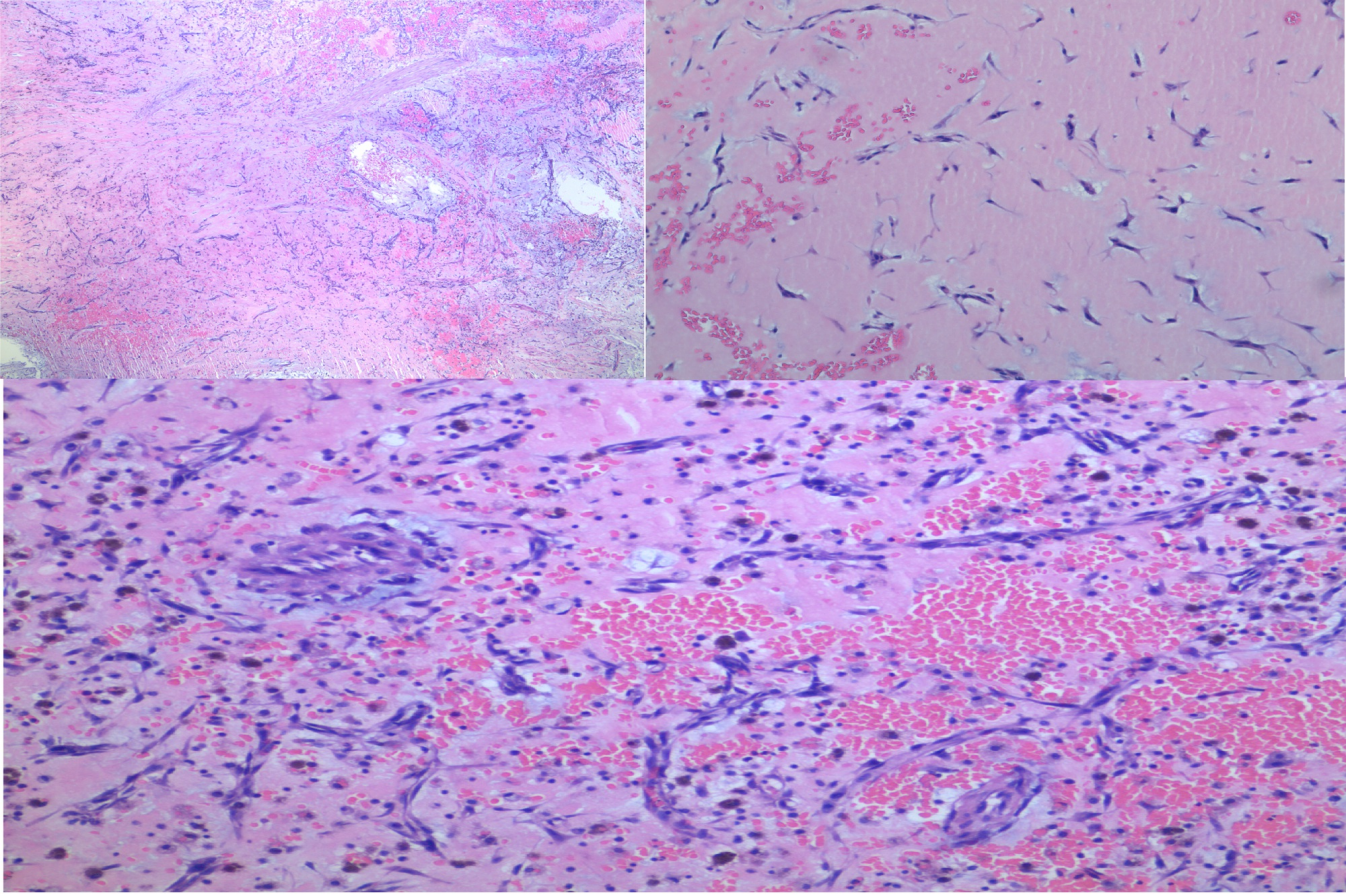
Tissue pathology showed spindle and satellite cells within a prominent myxoid matrix, a few scattered associated lymphocytes, and patchy foci of intraparenchymal hemorrhage

Chest X-ray showed no acute process. Due to concern of cerebrovascular accident, computed tomography (CT) of the brain with perfusion scan showed no hemorrhage or infarction (Figure 2). CT angiogram of the head and neck with contrast revealed 50% stenosis of left proximal internal carotid artery and no stenosis in intracranial vessels. Brain magnetic resonance imaging (MRI) confirmed multiple small acute infarcts involving bilateral posterior cerebral arteries distribution (Figure 3). Further evaluation included transthoracic echocardiography that showed a normal left ventricular ejection fraction of 60-65% and a large mobile mass in the left atrium measuring 3.5 x 2 cm that intermittently projected through the mitral valve. Transesophageal echocardiography confirmed these findings, and showed a normally appearing left atrial appendage with no thrombus formation (Video 1, Video 2). Bubble study showed no right to left shunt across the interatrial septum (Video 3). The patient underwent a successful left atrial myxoma resection with ligation of left atrial appendage. Intraoperatively, the left atrial mass appeared to be gelatinous and friable, and was attached to the lower atrial wall by a stalk. Care was taken to keep the mass intact during surgical excision. The stalk was then resected from the muscular wall of the left atrium including a margin of normal tissue. Pathology report showed spindle and satellite cells within a prominent myxoid matrix, a few scattered associated lymphocytes, patchy foci of intraparenchymal hemorrhage, with no malignancy identified, confirming the diagnosis of atrial myxoma (Figure 4).

**Video 1:** Transesophageal echocardiography showing a large left atrial mass intermittently projecting through mitral valve during diastole (red arrow is pointing towards mitral valve)

**Video 2:** Transesophageal echocardiography showing a normally appearing left atrial appendage with no thrombus formation (green asterisk indicates left atrial appendage)

**Video 3:** Transesophageal echocardiography with a bubble study on showing no right to left shunt across interatrial septum (green asterisk indicates left atrium; red asterisk indicates right atrium; note saline bubbles filling right atrium)

## Discussion

Epidemiology

Primary tumors of the heart are exceedingly rare, with a reported incidence of 0.0017-0.03% in autopsy series, and often represented by a myxoma in 50% of cases in the adult population. Metastatic tumors of the heart are 30 times more frequent [3]. It is estimated that 75% of cardiac myxomas arise within the left atrium. Other locations include the right atrium in 23% of cases and the ventricular cavity in 2% of cases [7]. This tumor affects mainly middle-aged female patients with a female-to-male ratio of 2 to 1 [6].

Clinical manifestations 

Cardiac myxoma is usually an isolated finding, although 10% of cardiac myxomas are associated with genetic syndromes. Carney complex is an autosomal dominant condition that presents with multiple endocrine and non-endocrine tumors including atrial myxoma. It has two subsets named with acronyms: LAMB syndrome, which is associated with lentigines, atrial myxoma, mucocutaneous myxomas, and blue nevi; and NAME syndrome, which presents with nevi, atrial myxoma, myxoid neurofibromas, and ephelides (freckles) [8].

Cardiac myxoma has a wide range of presentations that can be divided into embolic, obstructive, and constitutional which are summarized in Table 1. Our patient did not have previous constitutional or obstructive symptoms, and cardioembolic stroke was the initial presentation.

**Table 1:** Clinical manifestations of atrial myxoma

	Left-sided atrial myxoma	Right-sided atrial myxoma
Constitutional Symptoms	Fever, fatigue, myalgia, and weight loss. Pathogenesis: due to produced inflammatory cytokines like interleukin 6 and other acute phase reactants like C-reactive protein	
Obstructive Symptoms	Dyspnea with exertion, orthopnea (30%), paroxysmal nocturnal dyspnea, and pulmonary edema. On physical exam: “tumor plop" may be characteristically heard early in diastole (22%). Pathogenesis: due to mitral valve obstruction or regurgitation, left-sided heart failure, and secondary pulmonary hypertension	Dyspnea with exertion, pedal edema, hepatomegaly, and ascites. On physical exam: diastolic murmur, similar to the "tumor flop," can sometimes be appreciated at the tricuspid region; in addition, prominent "a wave" in the jugular veins, can also be observed occasionally. Pathogenesis: due to tricuspid stenosis and right heart failure
Thromboembolic Symptoms	Systemic embolization [9]: Central nervous system (most common): transient ischemic accident, cardioembolic stroke; Retinal arteries: sudden loss of vision; Coronary arteries: myocardial infarction; Other systemic organ embolism: spleen, kidneys, adrenals, abdominal aorta, iliac and femoropopliteal arteries.	Pulmonary arterial embolism: hypoxia, tachycardia, or sudden death Systemic embolism if an atrial septal defect or a patent foramen ovale coexist

Two anatomic types of atrial myxoma have been described; a solid (ovoid) type and a friable (papillary) type. Heart failure symptoms are more common in solid myxomas, while neurologic and embolization events are more common in the papillary type. Interestingly, histologic features also exhibit some correlation with clinical presentation; evidence of complete excision as well as the presence of fibrosis and gamma bodies are more likely to be associated with obstructive rather than embolic symptoms. Additionally, intratumoral hemorrhage is more commonly found in the solid type as compared to the friable type, possibly due to the vulnerability of its tenuous blood supply [10].

Diagnosis

Patients with atrial myxoma usually have a normal cardiac examination. Occasionally, an early diastolic tumor plop (early diastolic low-pitched sound just after the S2 heart sound), or gallop rhythm (due to the presence of a third heart sound, giving a rhythm that resembles the gallop of a horse) can be heard on auscultation [4,11]. However, most cardiac myxomas are found incidentally on echocardiography. Cardiac myxoma appears as a mobile mass attached to the endocardial surface by a stalk, usually arising from the interatrial septum at the region of fossa ovalis [12]. Cardiac magnetic resonance could particularly be helpful if the echocardiography could not provide optimal visualization of the mass or in patients who cannot undergo a transesophageal exam. It plays an important role in characterizing the mass including the shape, density, and tissue signal intensity contributing to more accurate diagnosis [13,14]. Elevated erythrocyte sedimentation rate, C-reactive protein, and white blood cell count can be seen with cardiac myxoma. Blood culture would be considered if infected cardiac myxoma is questioned. Histopathological examination of cardiac myxoma reveals round, polygonal, or stellate cells with dense irregular nuclei, ingrained in abundant, amorphous, and myxoid stroma containing mucopolysaccharides. Cells can be arranged individually, or as parallel clusters, syncytial cords, tubular structures, or in perivascular cuffs. In our patient, the histopathological examination showed spindle and satellite cells within an abundant myxoid matrix.

Echocardiography should be performed in all patients with stroke or transient ischemic attack (TIA) to rule out any cardiac causes. In a cohort study that included 1862 patients with stroke or TIA, 1272 patients (68%) had at least one echocardiogram. Among the cohort of echocardiograms performed, 86% were unremarkable, 0.08% had an atrial myxoma, 0.2% had valvular vegetation, 0.9% had an intracardiac thrombus, and 5.2% had a patent foramen ovale. Although the vast majority of patients in this study had a normal echocardiogram, 14% had cardiac abnormalities. This highlights the importance of echocardiogram for secondary stroke prevention [15].

Treatment and prognosis

Timely surgical excision is indicated in all patients to prevent systemic embolization and other complications. Although cardiac myxoma is histologically a benign tumor, it has well-known malignant behaviors such as local relapse, local invasiveness, and distant metastasis. The potential for malignant transformation is controversial, but malignant sarcomas arising from cardiac myxoma recurrences have been reported in the literature [3,16-18].

Intraoperatively, care should be taken to avoid any dislodging of tumor material. Repair or replacement of mitral or tricuspid valves may be needed depending on myxoma location. The most common postoperative complications include atrial fibrillation, complete atrioventricular block requiring permanent pacemaker implantation, cerebrovascular accident, and ventricular septal defect after ventricular myxoma resection. Postoperative in-hospital mortality is rare and occurs in less than 1% of the cases. Cardiac myxoma is associated with a 3.3% recurrence rate five years after surgical removal [19]; however, the recurrence rate is 12% in familial forms and 22% in patients with an underlying genetic syndrome such as Carney complex. Therefore, complete surgical excision encompassing the full thickness of normal surrounding tissue is recommended to reduce the recurrence rate. Recurrence has been reported as late as 10 years after initial surgical resection, and generally occurs at the same location as the original myxoma [19]. Follow-up echocardiography should be done three months after surgical resection then annually to monitor tumor recurrence and evaluate cardiac function [20].

## Conclusions

This case report highlights the importance of a complete evaluation of stroke, including echocardiography. Patients with cardiac myxoma should undergo timely surgical excision to prevent serious complications secondary to embolization.
